# Ultrasonographic assessment of the renal size using a kidney length to vertebral body length ratio in cats

**DOI:** 10.3389/fvets.2022.887746

**Published:** 2022-08-03

**Authors:** Miryam Martinez, Marta Soler, Francisco G. Laredo, Eliseo Belda, Amalia Agut

**Affiliations:** ^1^Veterinary Teaching Hospital, University of Murcia, Murcia, Spain; ^2^ProtonVet Veterinary Radiology, Hampshire, United Kingdom; ^3^Department of Medicine and Surgery, University of Murcia, Murcia, Spain

**Keywords:** cats, kidneys, vertebral bodies, ultrasound, radiography

## Abstract

Ultrasonographic assessment of the renal size can provide useful clinical information, in combination with other ultrasonographic parameters. The aims of this study were to establish the agreement between the ultrasonographic and radiographic measurements of the kidneys (K) and vertebral bodies (L5 and L6), to establish an ultrasonographic measurement of kidney-to-vertebral body (L5 and L6) ratio to estimate the renal size in cats, and to assess the impact of age, body weight, sex, and gonadal status on the ultrasonographic measurements of the kidneys, vertebral bodies, and ratios. The vertebral bodies of L5 and L6 were chosen as they were easy to identify with ultrasonography (US) using the lumbosacral junction as a landmark, and they are not usually affected by vertebral anomalies. A total of 60 cats (19 intact males, 12 neutered males, 17 intact females, and 12 neutered females) were included in the study. The cats were divided into three age groups (<7 months, 7 months−7 years, and >7 years), two body weight categories (≤ 3.5 kg and >3.5 kg), and two sex and gonadal status groups (male and female, and intact and neutered, respectively). Measurements of the renal and vertebral body length were performed on the radiographic and ultrasonographic images. Two different ratios were obtained, namely, K/L5 and K/L6. There was no significant difference between the length of both kidneys and the length of the vertebral bodies of L5 and L6 on ultrasonographic or radiographic images. There was a good agreement between ultrasonographic and radiographic measurements of both kidneys and vertebral bodies. In conclusion, the kidney length to L6 length ratio obtained was 1.81 ± 0.20 (1.76–1.86), which was useful for evaluating the size of the feline kidney and was not influenced by the age, body weight, sex, or gonadal status.

## Introduction

Ultrasound is a useful imaging tool to assess the kidneys in cats, providing an exhaustive evaluation of the size, shape, and renal architecture (1–4). Renal disease is a common problem in cats, and there are many diseases described, such as acute or chronic kidney disease, feline infectious peritonitis, or neoplasia amongst others, which can cause a variation in the renal size (1–5). Therefore, ultrasonographic assessment of the renal size can provide useful clinical information, in combination with other ultrasonographic parameters.

A wide range of values for the ultrasonographic renal length (between 3.0 and 4.5 cm, up to 5.3 cm) has been previously reported (1, 2). In addition, previous studies have described that the renal length is influenced by different factors such as breed, age, body weight, sex, or gonadal status (2, 6–9).

Multiple ultrasonographic methods for assessing the renal size in cats and dogs have been studied using different anatomical landmarks. The aorta has been used as a reliable landmark for multiple ratio studies (10, 11), including the establishment of the kidney-to-aorta ratio in both cats and dogs (9, 12). However, some limitations have been reported as hydration or changes in systemic arterial pressure, which can modify the aortic luminal diameter (12). In contrast, the vertebral bodies have also been used as anatomical landmarks, with fewer factors that can alter their size (6, 13–16). The kidney-to-vertebral body ratio has been described as a useful method in clinical practice to assess the renal size in dogs (13).

Nevertheless, to the best of our knowledge, no studies have described a kidney-to-vertebral body ratio as an ultrasonographic method of estimating the renal size in cats.

The aims of this study were to establish the agreement between the ultrasonographic and radiographic measurements of the kidneys and vertebral bodies (L5 and L6), and an ultrasonographic measurement of kidney-to-vertebral body (L5 and L6) ratio to estimate the renal size in cats. Finally, we evaluated the impact of age, body weight, sex, and gonadal status on the ultrasonographic measurements of the kidneys, vertebral bodies, and left kidney length/L5 length (LK/L5), left kidney length/L6 length (LK/L6), right kidney length/L5 length (RK/L5), and right kidney length/L6 length (RK/L6) ratios.

We hypothesized that ultrasound is a good imaging method to measure the length of the kidney and vertebral bodies to establish a kidney-to-vertebral body ratio as a method of estimating the renal size in healthy cats.

## Materials and methods

### Study population

This prospective study included the kidney and vertebral measurements of ultrasonographic and radiographic images obtained from client-owned cats, which were presented to the Veterinary Teaching Hospital of the University of Murcia for routine abdominal ultrasonography (US) between July 2020 and November 2021. Data collected from each animal included breed, age, body weight, sex, and gonadal status.

The cats were included in the study when the non-renal disease was demonstrated based on history, physical examination, hydration status assessment, results of hematological, biochemical, and urine analyses, and absence of ultrasonographic abnormalities. In addition, the cats showed negative results for feline leukemia and immunodeficiency virus tests. Cats with a history of urinary tract disease or abnormalities detected on any of the screening tests were excluded from the study.

The cats were divided into three age groups (<7 months, 7 months−7 years, and >7 years) (16), two body weight categories (≤ 3.5 kg and >3.5 kg), and two sex and gonadal status groups (male and female, and intact and neutered, respectively). All the neutered cats were sterilized at least 1 year before the study.

### Radiographs

A ventrodorsal view of the abdomen was taken using a computed radiography (CR 30-X, Agfa Healthcare NV, Mortsel, Belgium) previously to the US exam.

An external calibration marker (metallic plate) was used for calibration in order to reduce the effects of magnification. The marker was located adjacent to the abdomen.

### Ultrasonography

The patients were examined after fasting for at least 12 h prior to US exam. The abdomen was clipped, the skin was cleaned, and an acoustic coupling gel was applied. The US exam was performed, while the cats were conscious.

**Figure 1:**
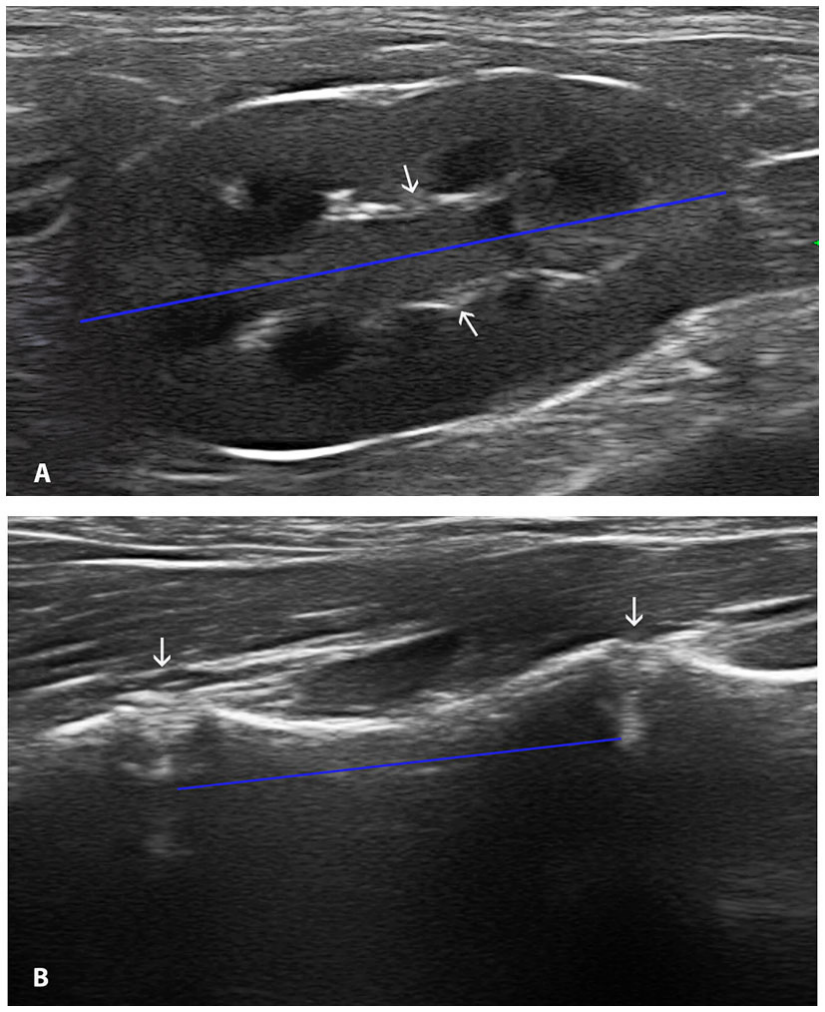
Midsagittal ultrasonographic image of the kidney passing through the hyperechoic ventral and dorsal branches of the renal pelvis visible as two hyperechoic parallel bars (arrows). The calipers were placed at the outside edge of each renal pole measuring the length (blue line) **(A)**. Longitudinal ultrasonographic image of L6. The calipers were placed at the artifact caused by the intervertebral disk (arrows) delimiting the extremes of the vertebral body (blue line) **(B)**.

All ultrasonographic examinations were performed by two operators (AA and MS) using a 4–13 MHz linear array transducer (Mylab Twice LA523, Esaote). The settings were adapted in order to possibly acquire the best image quality. The cats were positioned in dorsal recumbency to record the renal images and in right lateral recumbency to obtain the images of the vertebral bodies. The midsagittal plane of both kidneys was acquired, which is characterized by the appearance of two bright parallel bars formed by cross-sectioned pelvic diverticula (Figure 1A) (8). Longitudinal sections of L5 and L6 were also obtained. To identify the vertebral bodies of L5 and L6, the lumbosacral junction was visualized and then the transducer was moved cranially identifying in sequences L7, L6, and L5. The longitudinal plane of the vertebral body is characterized as a hyperechoic curvilinear line with acoustic shadow and a cranial and caudal gap (intervertebral disks) between the lines (Figure 1B).

### Data acquisition

Ultrasonographic and radiographic images were saved in a DICOM format. An image viewer (OSIRIX MD 12.0.3) was used to measure the kidneys and vertebral bodies in both imaging modalities. The images were displayed on a monitor (32Dell UP3216Q 4K). The same investigator (MM) performed all measurements using the image viewer to ensure consistency. The caliper size was 1 pixel.

On the ultrasonographic images, the maximal length of each kidney was measured from the cranial to the caudal pole through the renal pelvis, placing the calipers at the outside edge of each renal pole (Figure 1A). The maximum length of the vertebral bodies was measured using the artifact caused by the intervertebral disk for delimiting the extremes of the vertebrae (13) (Figure 1B). Each measurement was obtained three times.

**Figure 2:**
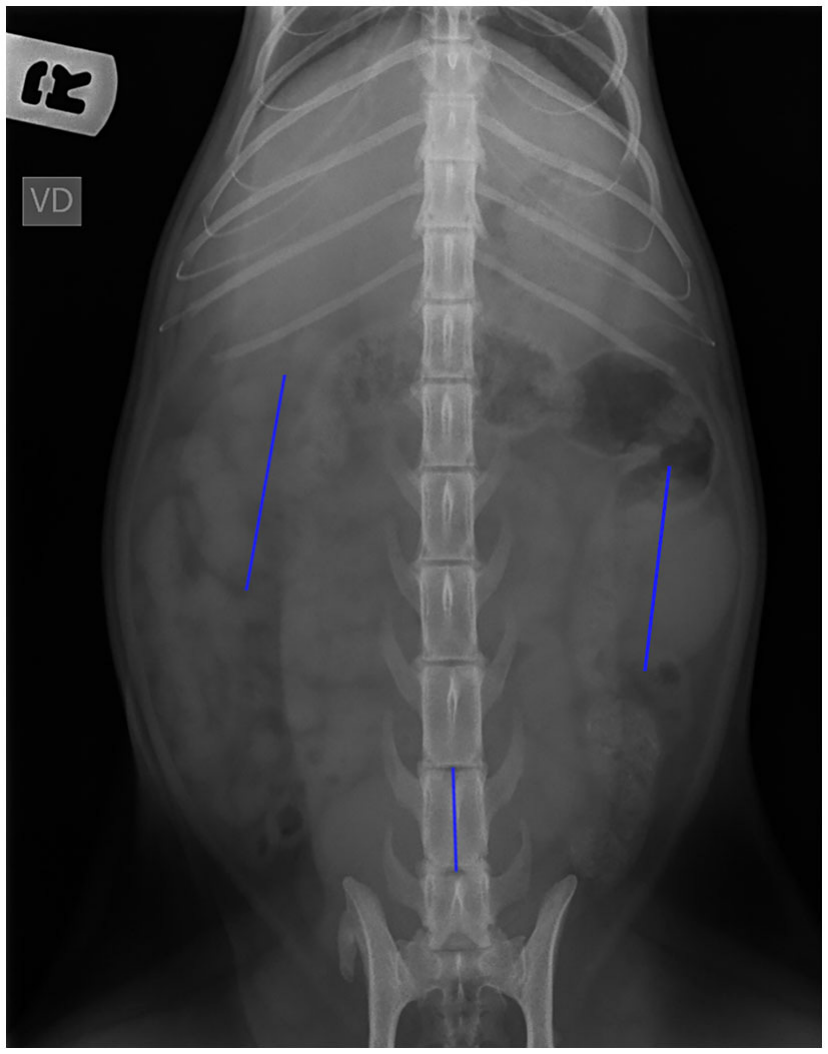
Ventrodorsal radiographic view of the abdomen. Note the position of the calipers to measure the length of each kidney and the vertebral bodies.

The radiographic measurements of both kidneys were obtained in the ventrodorsal view of the abdomen. The length of both kidneys and the length of the vertebral bodies of L5 and L6 were recorded. The radiographic kidney length was considered the maximum distance between cranial and caudal poles of the kidneys. The body length of the L5 and L6 was measured from the cranial to the caudal endplates. The images were calibrated based on the measurements of a metallic plate that was placed on the side of the image (Figure 2). Each measurement was obtained three times.

Four different ratios were obtained on each imaging modality, left kidney length/L5 length (LK/L5), left kidney length/L6 length (LK/L6), right kidney length/L5 length (RK/L5), and right kidney length/L6 length (RK/L6).

### Statistical analysis

Statistical tests were performed using R version 4.0.3 (R Core Team 2020). All data obtained from ultrasound and radiographs were tested for normal distribution using the Shapiro–Wilk or Kolmogorov–Smirnov test analysis depending on the amount of data. The homoscedasticity was assessed using the Fligner–Killeen test.

For the normally distributed data, the descriptive statistics used were mean, standard deviation (SD), and maximum and minimum values.

To assess the differences between each of the three measurements obtained in each variable, a repeated-measures ANOVA was used. The Bland–Altman analysis was used to assess the limit of agreement between the ultrasonographic and radiographic measurements of both kidneys and vertebral bodies of L5 and L6. An agreement was considered good if 95% of the absolute differences were within two SDs (SD ± 1.96).

A two-sample *t*-test was used to determine whether there were significant differences between radiographic and ultrasonographic measurements of the renal dimensions and the size of the vertebral bodies of L5 and L6, as well as the differences between the radiographic and ultrasonographic left vs. right kidney size and L5 vs. L6 dimensions.

The effect of age, body weight, sex, and gonadal status in the dimensions and ratios of the kidneys and vertebral bodies (LK/L5, LK/L6, RK/L5, and RK/L6) was analyzed with an independent *t*-test or one-way ANOVA. The independent *t*-test was used in variables of two levels such as body weight, sex, and gonadal status. The one-way ANOVA was used when the variable had three levels (age). All statistical analyses were considered significant if *P* < 0.05.

## Results

A total of 60 cats (19 intact males, 12 neutered males, 17 intact females, and 12 neutered females) were included in the study. The cats ranged from 1.5 months to 14 years old (median age was 2 years). Notably, 10 cats were <7 months old, 36 cats were between 7 months and 7 years old, and 14 cats were more than 7 years old. The mean body weight for the population was 4.02 kg (range 0.7–7.9 kg). Out of 60 cats, 25 cats weighed ≤ 3.5 kg and 35 cats weighed more than 3.5 kg.

### Ultrasonographic measurements

The kidneys and vertebral bodies of L5 and L6 were identified by ultrasound in all cats. There were no significant differences between the 3 measurements obtained on each variable based on the repeated-measures ANOVA, and the mean of the three measurements was used.

The ultrasonographic measurements of the length of both kidneys and the vertebral body of L5 and L6 are shown in Table 1. No statistical difference was found between the length of both kidneys nor between the length of vertebral bodies of L5 and L6. As there were no statistical differences between the length of the right and left kidneys, the ratios were also calculated including the values of both kidneys (K/L5 and K/L6 ratios). Table 2 summarizes the values of the kidney length to L5 and L6 ratios. No statistical differences were found between K/L5 and K/L6 ratios.

**Table 1:** Ultrasonographic (US) and radiographic (XR) measurements of renal and vertebral bodies length (cm).

	**US**	**XR**
	**Mean ± SD** **Range**	**Mean±SD** **Range**
Left kidney	3.77 ± 0.40 (2.74–4.60)	4.25 ± 0.53 (3.30–5.49)
Right kidney	3.9 ± 0.41 (3.06–4.82)	4.36 ± 0.54 (3.42–5.75)
Vertebral body L5	2.16 ± 0.23 (1.24–2.66)	2.21 ± 0.31 (1.12–3.01)
Vertebral body L6	2.14 ± 0.27 (1.24–2.92)	2.18 ± 0.30 (1.10–2.90)

**Table 2:** Ultrasonographic (US) and radiographic (XR) ratios of kidney to vertebral bodies length.

	**US**	**XR**
**Ratios**	**Mean ± SD** **Range**	**Mean ± SD** **Range**
LK/L5	1.91 ± 0.17 (1.61–2.48)	1.76 ± 0.18 (1.41–2.21)
LK/L6	1.93 ± 0.19 (1.57–2.62)	1.78 ± 0.20 (1.42–2.41)
RK/L5	1.97 ± 0.18 (1.61–2.60)	1.82 ± 0.20 (1.44–2.46)
RK/L6	1.99 ± 0.21 (1.69–2.75)	1.84 ± 0.21 (1.51–2.47)
K/L5	1.79 ± 0.18 (1.74–1.84)	1.94 ± 0.17 (1.89–1.98)
K/L6	1.81 ± 0.20 (1.76–1.86)	1.96 ± 0.20 (1.9–2.02)

LK, Left kidney; RK, Right kidney; K, Both kidneys.

### Radiographic measurements

The abdominal radiograph could not be taken on 3 animals due to a lack of patient cooperation. Both kidneys were not identified on the radiograph of one cat (1.5 months old) due to the loss of serosal detail given the immature status of the patient and the presence of physiological ascites. The right kidney was not clearly visualized in the other five cats due to superimposition with other abdominal structures. The measurements of the kidney in those cats were not included in the study. A total of 56 LK and 51 RK were measured. There were no significant differences between the 3 measurements obtained on each variable based on the repeated-measures ANOVA, and the mean of the three measurements was used.

The radiographic measurements of the length of both kidneys and the vertebral body of L5 and L6 are shown in Table 1. The values of the ratios for both kidneys to L5 and L6 are summarized in Table 2.

There was no significant difference between the length of both kidneys and the length of the vertebral bodies of L5 and L6. As there were no statistical differences between the length of the kidneys, the ratios were calculated including both kidneys (K/L5 and K/L6 ratios) (Table 2). No significant differences were found between K/L5 and K/L6 ratios.

### Agreement between ultrasonographic and radiographic measurements

**Figure 3:**
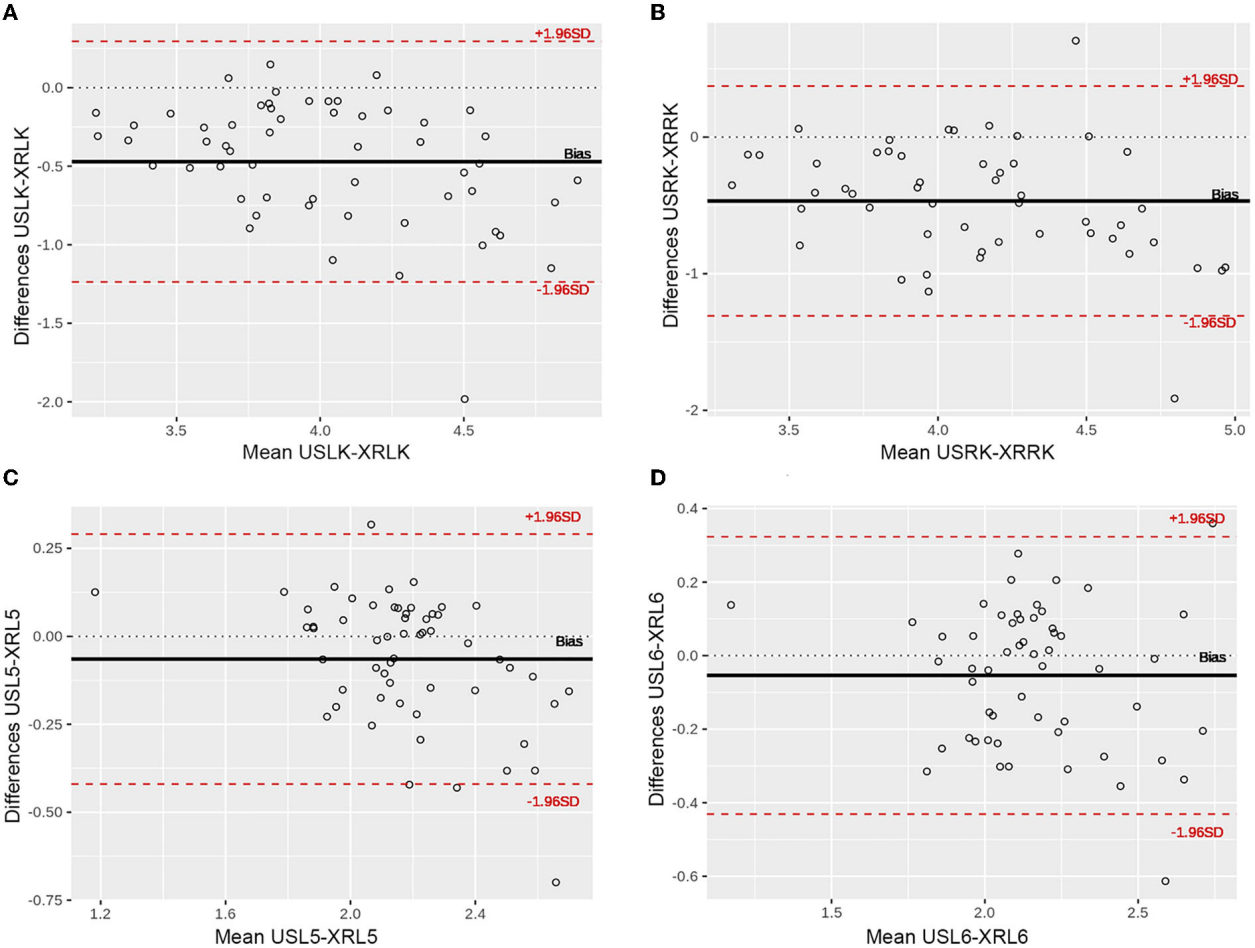
Bland–Altman plots illustrating the comparison of the measurements obtained with ultrasound (US) and radiograph (XR). The differences between the two measurements are plotted against the averages of the differences. **(A)** Left kidney; **(B)** Right kidney; **(C)** Vertebral body L5; **(D)** Vertebral body L6. Note that more than 95% of differences are within two standard deviations, showing a good agreement between both methods. USLK, ultrasound left kidney; XRLK, radiograph left kidney; USRK, ultrasound right kidney; XRRK, radiograph right kidney; USL5, ultrasound L5; XRL5, radiograph L5; USL6, ultrasound L6; XRL6, radiograph L6.

The Bland–Altman analysis revealed a good agreement between ultrasonographic and radiographic measurements of both kidneys and vertebral bodies. However, the radiographic measurements were slightly bigger than the ultrasonographic measurements for the kidneys and vertebral bodies. Therefore, mean differences in the Bland–Altman analysis were negative for both kidneys and vertebral bodies (Figure 3).

### Influence of the age, body weight, sex, and gonadal status in the kidneys and vertebral bodies length and ratios using ultrasound

**Figure 4:**
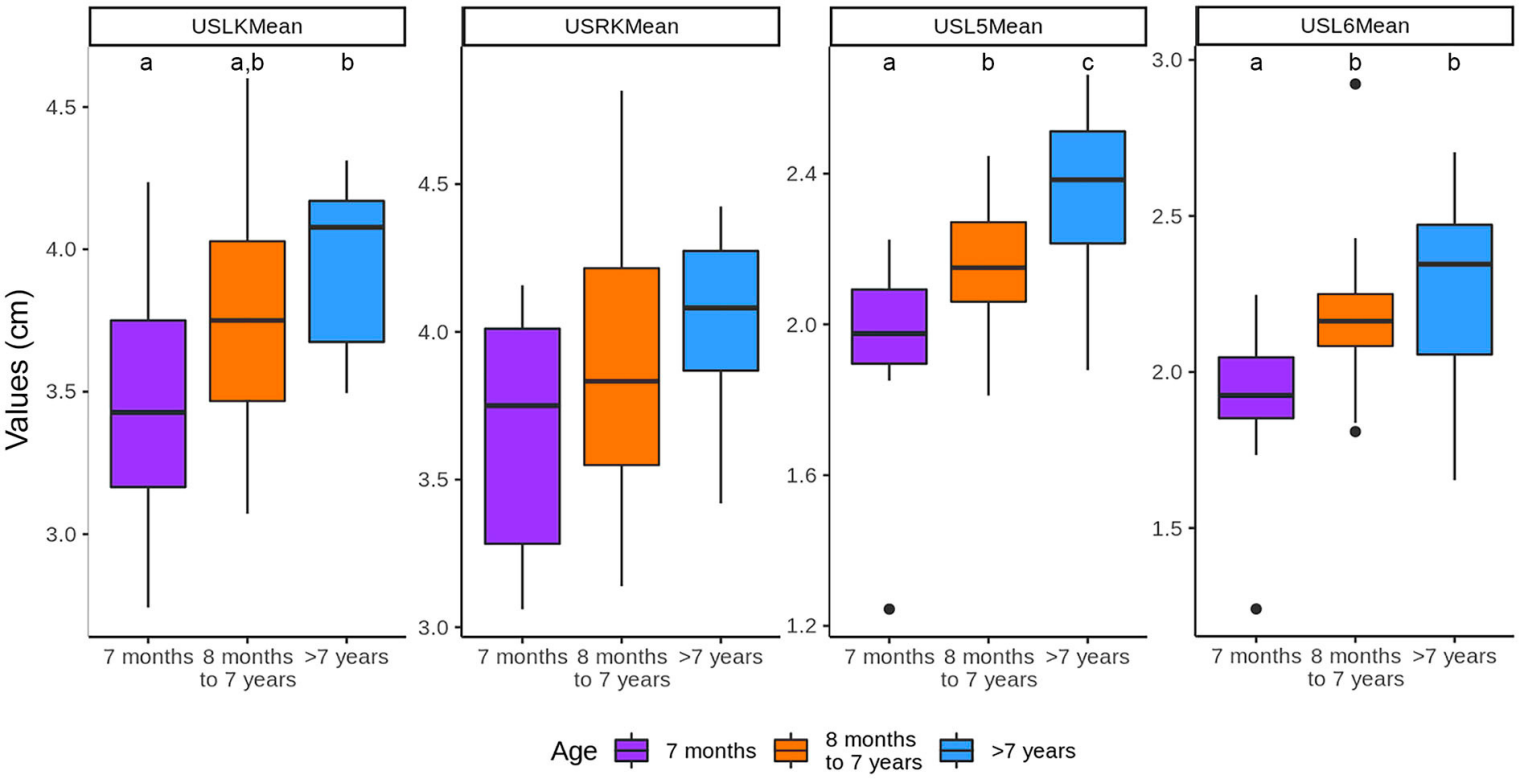
Influence of the age in the length of the kidneys and vertebral bodies. ^a−*c*^Within each group, values with different letters are statistically different (*P* < 0.05). USLK, ultrasound left kidney; USRK, ultrasound right kidney; USL5, ultrasound L5; USL6, ultrasound L6.

The length of both kidneys increased with the age of the cats. The left kidney values were lower (P < 0.05) in the group of <7 months (3.47 ± 0.45 cm) compared with >7 years (3.96 ± 0.28 cm). However, there were no significant differences between the three age groups for the right kidney length. The length of both vertebral bodies also increased with the age. There was a difference (P <0.05) between the three groups of age in L5 and between the cats aged <7 months with the other two groups of age in L6 (Figure 4).

**Figure 5:**
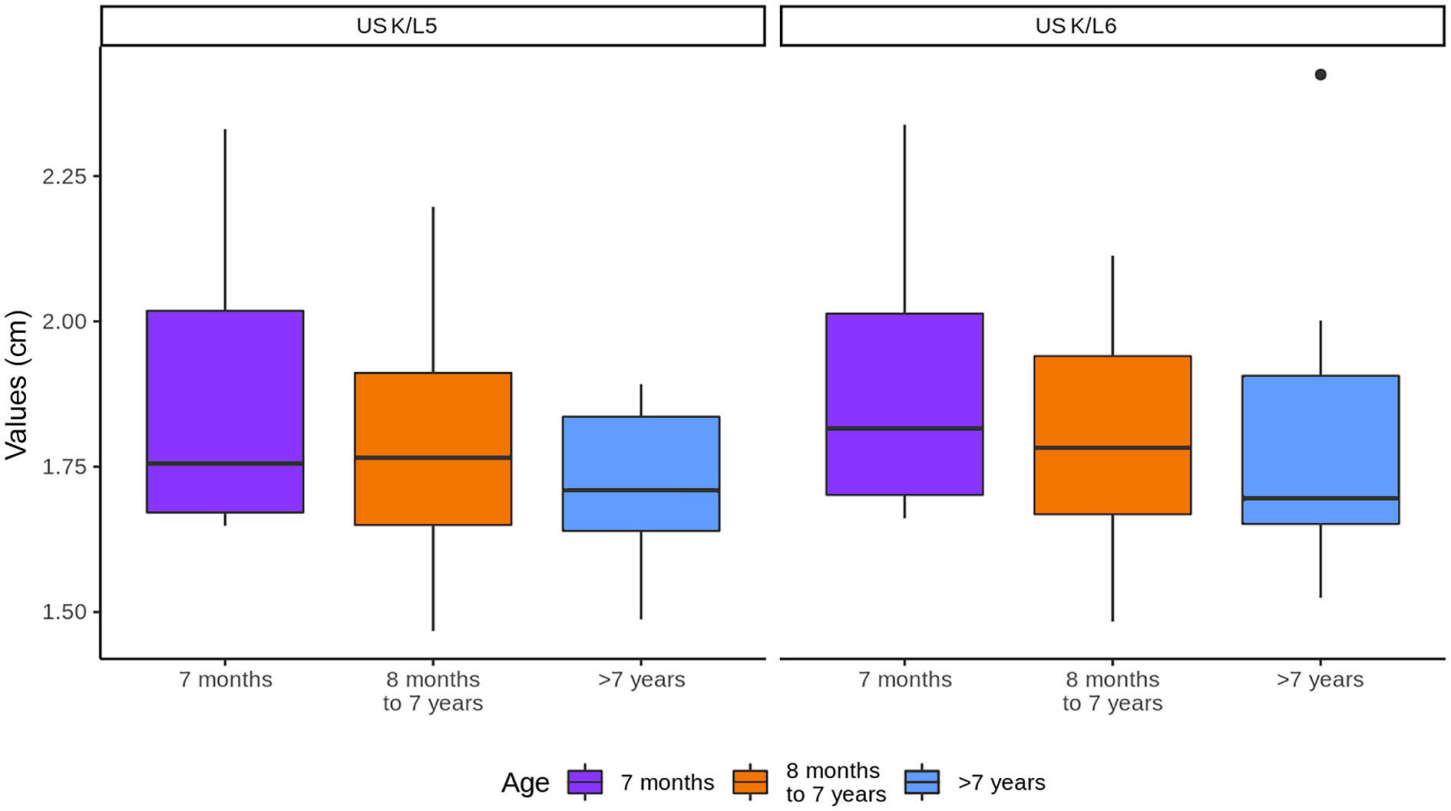
Influence of age in the kidneys (K) to L5 and L6 ratios. Note that K represents the values of both kidneys.

The age did not have an influence on K/L5 and K/L6 ratios (Figure 5).

**Figure 6:**
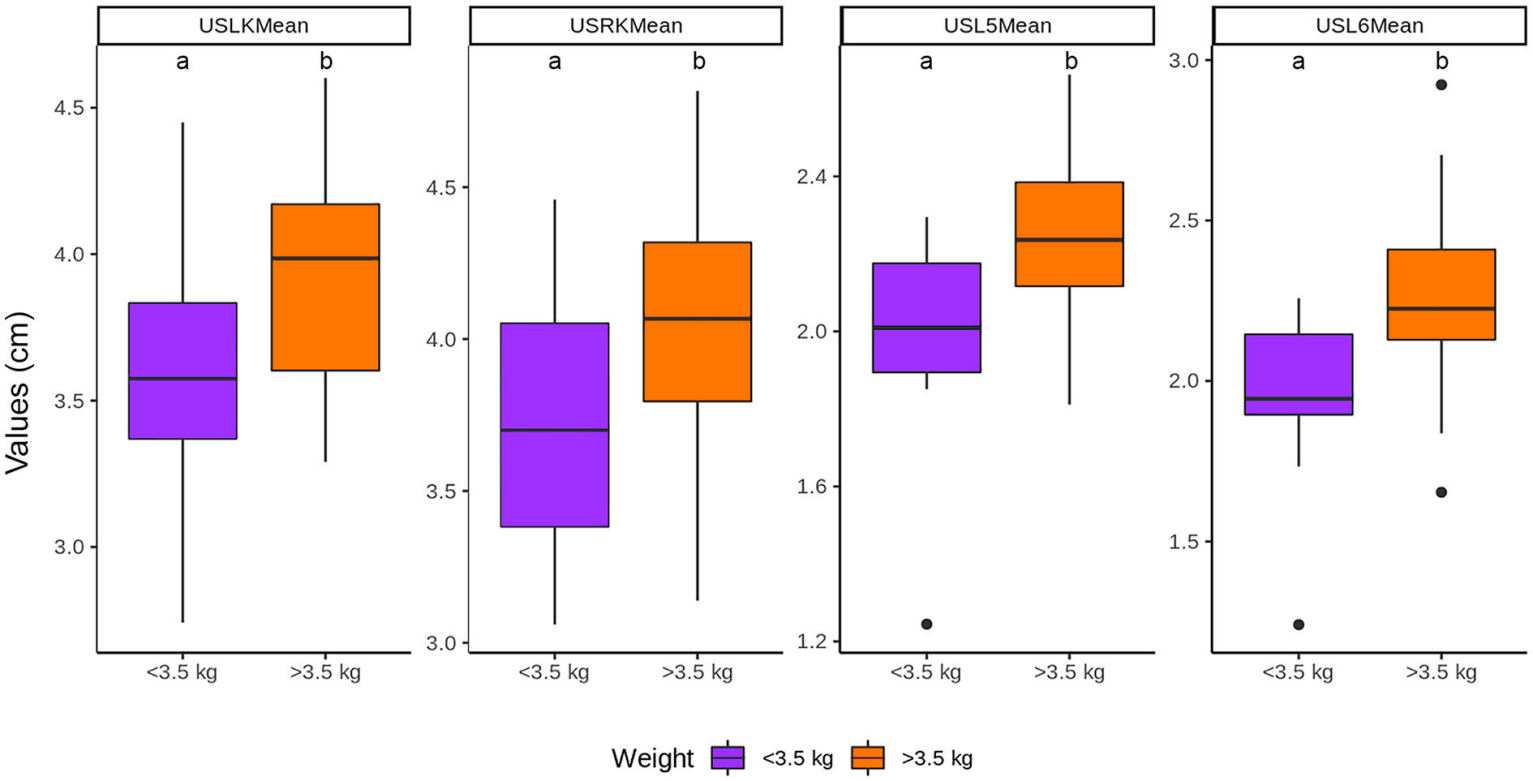
Influence of the body weight in the length of the kidneys and vertebral bodies. ^a, b^Within each group, values with different letters are statistically different (*P* < 0.05). USLK, ultrasound left kidney; USRK, ultrasound right kidney; USL5, ultrasound L5; USL6, ultrasound L6.

The length of both kidneys and the vertebral bodies increased with the body weight, being longer (*P* < 0.05) in the group of >3.5 kg than in the group of ≤ 3.5 kg (Figure 6).

**Figure 7:**
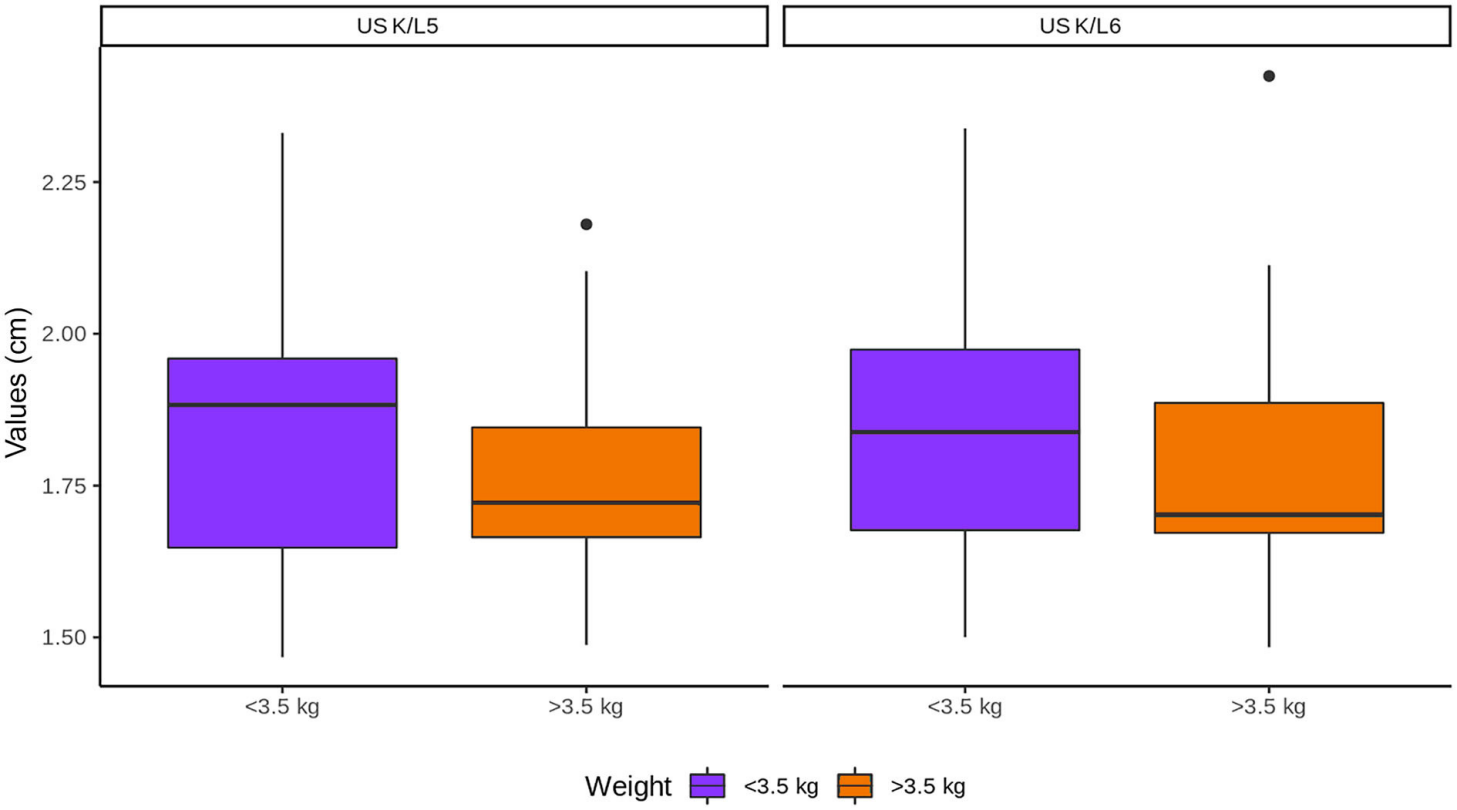
Influence of body weight in the kidneys (K) to L5 and L6 ratios. Note that K represents the values of both kidneys.

The body weight did not influence K/L5 and K/L6 ratios (Figure 7).

**Figure 8:**
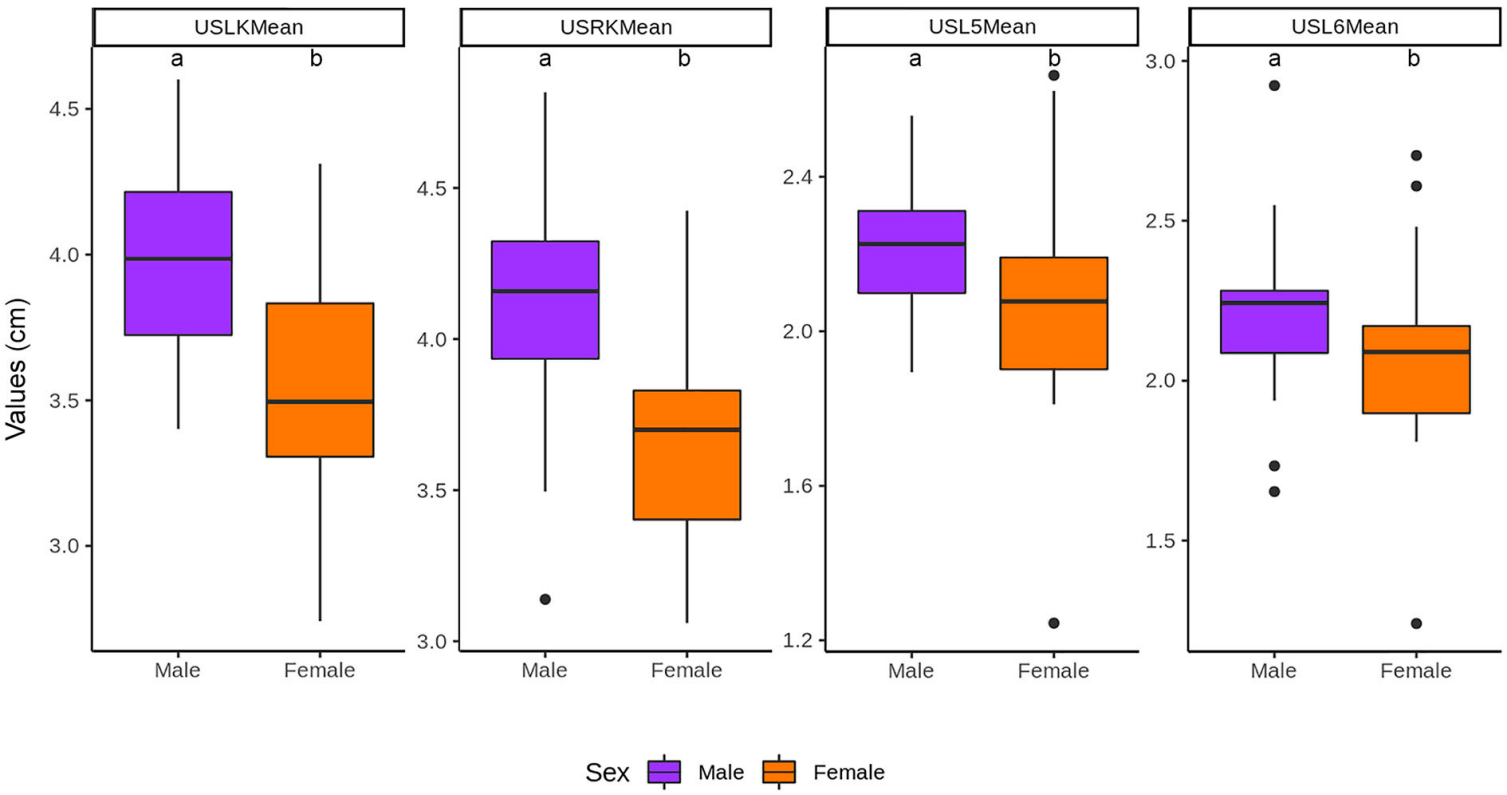
Influence of the sex in the length of the kidneys and vertebral bodies. ^a, b^Within each group, values with different letters are statistically different (*P* < 0.05). USLK, ultrasound left kidney; USRK, ultrasound right kidney; USL5, ultrasound L5; USL6, ultrasound L6.

**Figure 9:**
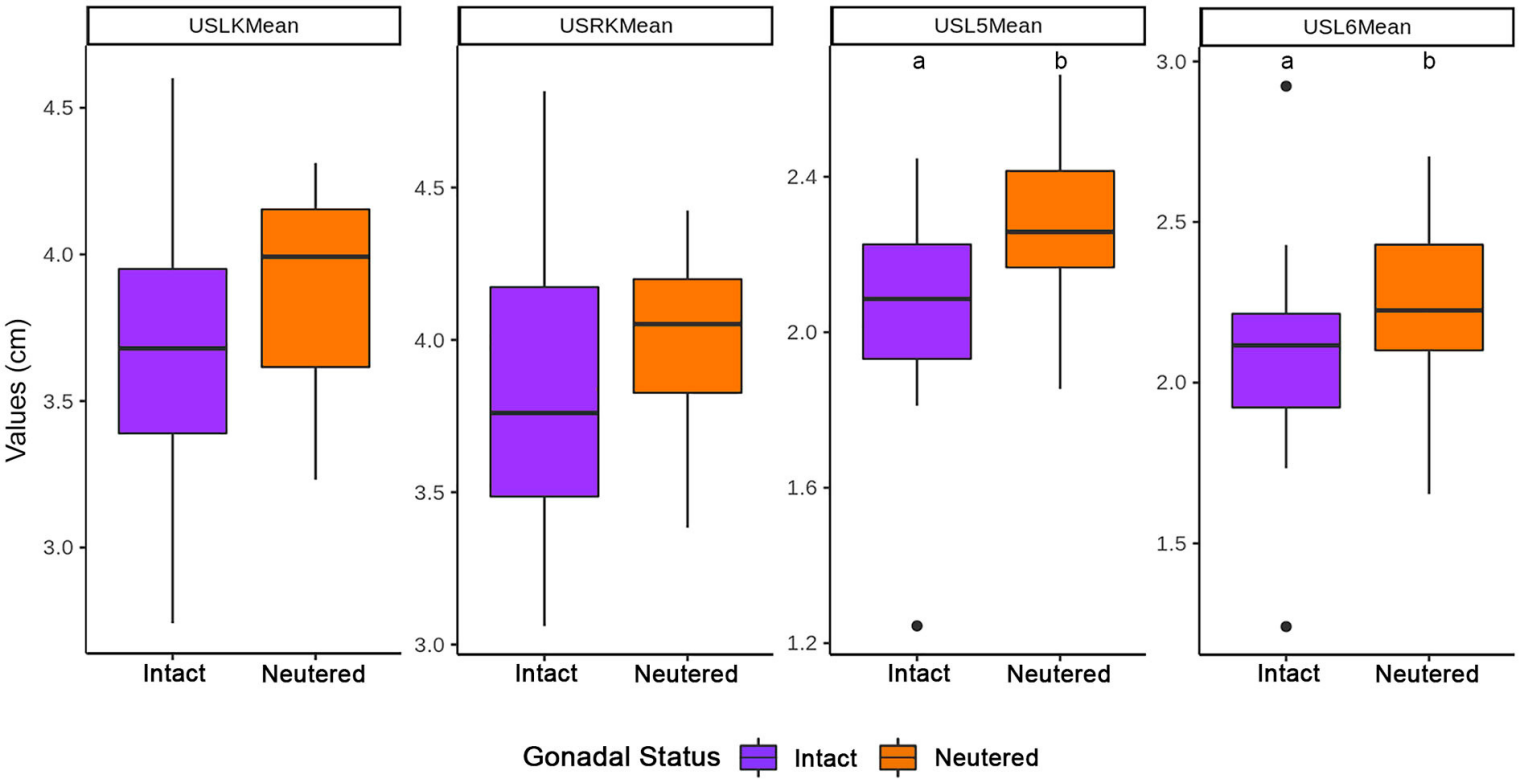
Influence of the gonadal status in the length of the kidneys and vertebral bodies. ^a, b^Within each group, values with different letters are statistically different (*P* < 0.05). USLK, ultrasound left kidney; USRK, ultrasound right kidney; USL5, ultrasound L5; USL6, ultrasound L6.

The length of the renal and vertebral bodies of male cats was longer (*P* < 0.05) than those of female cats (Figure 8). The length of the vertebral bodies was influenced by the gonadal status (*P* < 0.05), being longer in neutered cats. However, the renal length was not influenced by the gonadal status (Figure 9).

**Figure 10:**
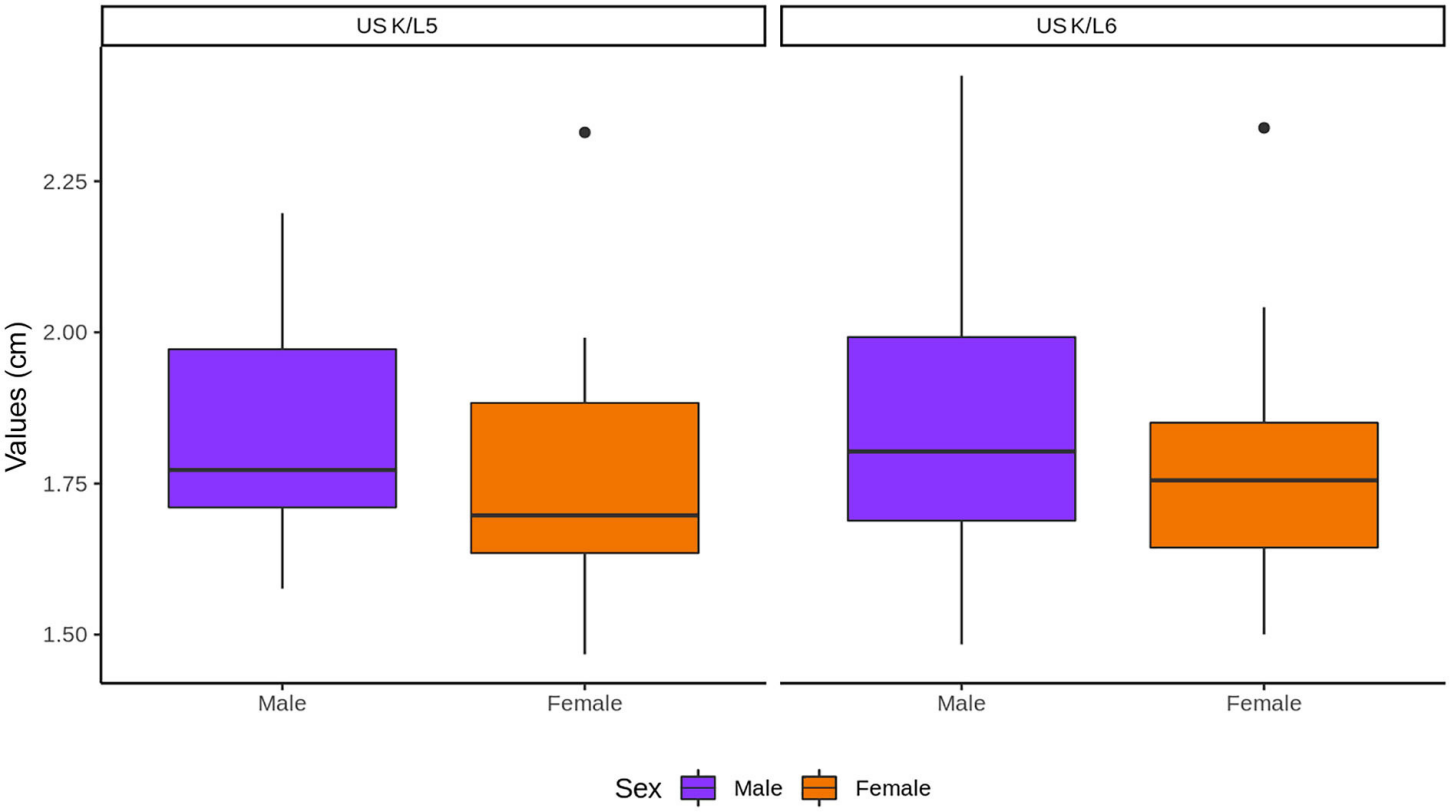
Influence of sex in the kidneys (K) to L5 and L6 ratios. Note that K represents the values of both kidneys.

**Figure 11:**
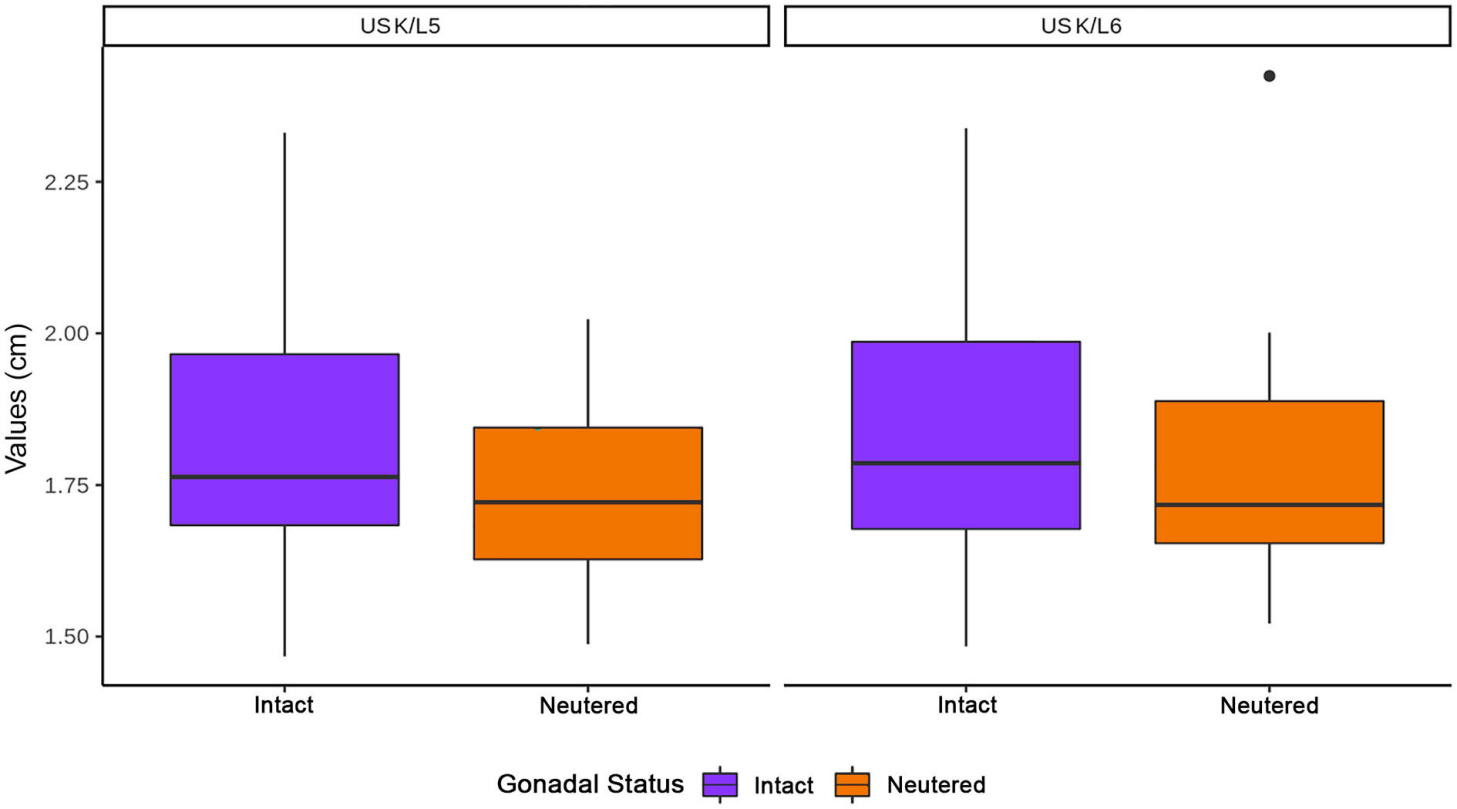
Influence of gonadal status in the kidneys (K) to L5 and L6 ratios. Note that K represents the values of both kidneys.

The sex and gonadal status did not influence K/L5 and K/L6 ratios, although the ratios were higher in males and intact cats (Figures 10, 11, respectively).

## Discussion

This study demonstrated a good agreement between ultrasonographic and radiographic measurements of both kidneys' and lumbar vertebral body's length. We detected an effect of age, body weight, sex, and gonadal status on the length of kidneys and lumbar vertebral bodies. However, there was no effect of these variables on K/L5 or K/L6 ratios. For this reason, ratios can be considered independent of patient's variables and likely to provide more useful information than simple linear dimension (17).

Previous ultrasonographic studies have reported that the normal size of the feline kidney varies between 3.0 and 4.3 cm and can reach 5.3 cm in length (1, 2, 5, 7, 18–20). This corresponds with our values of the mean renal length of the right kidney 3.9 cm ± 0.41 and the left kidney 3.77 cm ± 0.40.

In our study, no significant differences were found between the length of the right and left kidneys. This finding is consistent with another study using computed tomography (CT) (16). However, in other studies performed by ultrasound, the right kidney was significantly longer than the left kidney (7, 9). Based on the absence of significant differences between the lengths of both kidneys, all the renal measurements were used to obtain kidney/lumbar vertebral body ratios (K/L5 and K/L6).

The renal size in relation to the vertebral body length has been studied in cats using radiographs and CT (6, 16). The radiological method has been adapted for use in US to assess the kidney size in dogs (13). To the best of our knowledge, ultrasonographic evaluation of the kidney size in relation to the vertebral body in cats has not yet been studied. It can be a reliable, quick, and simple method to estimate the renal size in cats of routine use in veterinary practice.

In abdominal radiography and CT, the length of the kidney is compared with the length of the second lumbar vertebra (L2) (6, 16). However, in ultrasound, it is difficult to scan L2 because it is more cranial and the costal arches hinder the progression of the transducer. For this reason, the last lumbar vertebrae (L5, L6, and L7) are used (13). We excluded the use of L7 because it is shorter than L5 and L6 (21) and may be affected by vertebral anomalies (22). The vertebral bodies of L5 and L6 were easy to identify in all cases by landmark to the lumbosacral junction. There was no significant difference between the lengths of L5 and L6.

In this study, we detected an effect of age, body weight, sex, and gonadal status on the length of the kidneys and the lumbar vertebral bodies.

The length of the kidneys increased with the age of the cats (8). This increase was significant for the left kidney. Similar results have been described in people, where the relationship between age and kidney is linear until 16 years of age. Subsequently, the size of the kidney is fairly constant with a slight predisposition to decrease with time (23). We believe that the differences between the group of <7 months compared with >7 years could be related to a more immature status of the organ in the group of <7 months. On the contrary, in another study using CT, the size of feline kidneys was similar in all ages. The differences between both studies may be because our population of cats was larger (60 cats vs. 27 cats) and because the number of cats in each group was different.

In contrast, the length of the vertebral bodies also increased with the age with differences (P < 0.05) between cats aged <7 months and the other two groups in L6. This may be due to the fact that the age of the closure of the vertebral endplates has been reported between 7 and 11 months in cats (24).

In our study, the length of the kidney (8) and vertebral bodies increased with the body weight, as previously reported. In addition, the group of older cats (>7 years) was the ones with a heavier body weight. It is surprising that the vertebral body length is significantly influenced by the body weight. However, this could have also been influenced by the age of the cats, considering that the older cats were the heaviest ones. In contrast, it has been suggested that retroperitoneal and hilar fat can influence the measurement of kidney size in cats (25). This fact could be another reason whereby the kidneys were longer in cats with higher body weight.

As previously reported in other studies, the male cats had longer (*P* < 0.05) kidneys and vertebral bodies (7, 8, 20). This could be influenced by the body weight, as the males had higher body weights compared to females. On the contrary, there are other studies where the renal size between sexes was not different (6, 16).

In our study, neutered cats showed longer kidneys and vertebral bodies than intact animals, which was considered significant (*P* < 0.05) for the vertebral bodies. It has been previously described that sex hormones (oestrogens and testosterone) have a positive effect on the renal size within respective sexes (6). Therefore, the intact animals showed longer kidneys. However, there are other studies where neutered cats regardless of the sex tend to have longer kidneys without significant differences (9), as previously described in our study. In contrast, it has been reported that neutered cats between 7 weeks and 7 months had a significantly delayed physeal closure and, as a consequence, longer bones compared with intact cats (26). In our study, the cats were sterilized for more than 1 year before the study; however, we did not know exactly at what age the cats were sterilized. This could be an explanation for why the vertebral bodies were significantly longer in neutered cats in our study.

In this study, the K/L5 and K/L6 ratios were not influenced by any of the factors studied and there were no differences between them. Therefore, both ratios would be valuable to assess the ultrasonographic kidney size independently of age, body weight, sex, and gonadal status. Therefore, the ratios provide more useful information compared to a single linear dimension (27). We recommended using the K/L6 ratio because the vertebral body of L6 was easier to identify compared to L5, as previously described in dogs (13).

This study has some limitations. We use the vertebral body as a landmark to establish a ratio, and changes at the vertebra could interfere to establish the ultrasonographic vertebral length. A study established that spondylosis is a common disease in cats, with a higher prevalence in older animals and with a more severe degree of spondylosis affecting the lumbar or lumbosacral regions (28). However, it has been reported in dogs that the difficulty was never substantial enough to prevent measurement (13). In our study, spondylosis was not detected in any of the animals.

In our study, we did not have breed variability, with only three breeds included and the vast majority being domestic shorthair cats. Therefore, we could not assess if the K/L6 ratio could be influenced by the cat's breed. However, another recent study did not show statistically significant differences in renal dimensions comparing different feline breeds (7). Another limitation was that the number of immature cats was not elevated, and this could affect our results. Further studies including more immature cats would be helpful to investigate if the immature status of the patients could affect the K/L6 ratio. Finally, we did not have the gross anatomy to assess the actual size of the kidneys and vertebral body. This was not possible since the cats were healthy or presented minor conditions, and none of them was euthanised after the radiographic and ultrasonographic study.

In conclusion, the ultrasonographic kidney length to L6 length ratio is a useful and practical method for evaluating the size of the feline kidney and there is a good agreement with the radiographic values. The kidney length to L6 length ratio obtained was 1.81 ± 0.20 (1.76–1.86), which was not influenced by age, body weight, sex, or gonadal status of the cats. As an alternative, the kidney length to L5 length ratio can be obtained, if it is not possible to determine the kidney length to L6 length ratio, with a value of 1.79 ± 0.18 (range 1.74–1.84). Therefore, the results of this study can be used as reference values for normal ultrasonographic renal dimensions in cats. However, this ratio must be carefully interpreted together with other parameters such as clinical signs, test results, and other ultrasonographic parameters, and futher studies are needed to assess the usefulness of this ratio in cats with renal disease.

## Data availability statement

The original contributions presented in the study are included in the article/supplementary material, further inquiries can be directed to the corresponding author.

## Ethics statement

The animal study was reviewed and approved by Animal Care and Ethics Committee of the University of Murcia. Written informed consent was obtained from the owners for the participation of their animals in this study.

## Author contributions

AA and MM contributed to conception and design of the study. AA and MS were involved in the acquisition of the images. MM performed the measurements of the study and drafting of the manuscript. FL and EB performed the analysis and interpretation of the data. AA revised the manuscript. All authors contributed to the article and approved the submitted version.

## Conflict of interest

The authors declare that the research was conducted in the absence of any commercial or financial relationships that could be construed as a potential conflict of interest.

## Publisher's note

All claims expressed in this article are solely those of the authors and do not necessarily represent those of their affiliated organizations, or those of the publisher, the editors and the reviewers. Any product that may be evaluated in this article, or claim that may be made by its manufacturer, is not guaranteed or endorsed by the publisher.
